# Importance of the origin of mesenchymal (stem) stromal cells in cancer biology: “alliance” or “war” in intercellular signals

**DOI:** 10.1186/s13578-021-00620-6

**Published:** 2021-06-10

**Authors:** Noemi Eiro, Maria Fraile, Silvia Fernández-Francos, Rosario Sánchez, Luis A. Costa, Francisco J. Vizoso

**Affiliations:** 1 Unit Research, Fundación Hospital de Jove, Avda. Eduardo Castro 161, 33290 Gijón, Asturias Spain; 2 Department of Surgery, Fundación Hospital de Jove, 33290 Gijón, Asturias Spain

**Keywords:** Tumor microenvironment, Uterine mesenchymal stem cell, Cancer associated fibroblasts, Macrophage associated cancer

## Abstract

Mesenchymal stem cells (MSCs) play a central role in the intercellular signaling within the tumor microenvironment (TME), exchanging signals with cancer cells and tumor stromal cells, such as cancer-associated fibroblasts and inflammatory mononuclear cells. Research attributes both pro-tumor and anti-tumor actions to MSCs; however, evidence indicates that MSCs specific effect on the tumor depends on the source of the MSCs and the type of tumor. There are consistent data proving that MSCs from reproductive tissues, such as the uterus, umbilical cord or placenta, have potent anti-tumor effects and tropism towards tumor tissues. More interestingly, products derived from MSCs, such as secretome or extracellular vesicles, seem to reproduce the effects of their parental cells, showing a potential advantage for clinical treatments by avoiding the drawbacks associated with cell therapy. Given these perspectives, it appears necessary new research to optimize the production, safety and antitumor potency of the products derived from the MSCs suitable for oncological therapies.

## Introduction

The worldwide number of cancer patients is expected to increase from 14 million in 2012 to more than 19 million in 2025 (http://www.wcrf.org/cancer_statistics/world_cancer_statistics.php). Despite all the improvements made in its prevention, diagnosis and treatment, cancer still supposes and will be an important cause of morbidity and mortality. In addition to this, we have to consider the adverse effects of treatments such as chemotherapy, radiotherapy, hormonal therapy and immunotherapy. For all these reasons, it is time to try new therapeutic alternatives through the new emerging paradigms of science and medicine. On the lookout for that, we probably have to start from the basis of cancer. Cancers are the result of a complex interaction between cancer cells and their tumor microenvironment (TME), which comprises extracellular vesicles, bioactive soluble molecules, cellular matrix and, mainly, stromal cells. The interactions between tumor cells and the non-malignant stromal cells display a key role for the TME, and consequently, a crucial gear for the pathophysiology of cancer. Stromal cells are key structural and functional elements in all human carcinomas, comprised of immune cells (macrophages, neutrophils, mast cells, T- and B-lymphocytes), fibroblasts, endothelial cells, pericytes and mesenchymal (stem) stromal cells (MSCs) [1]

MSCs, non-hematopoietic and multipotent cells, were first described by hematologist A. Friedenstein and his collaborators, approximately 50 years ago, as a rare population (1 in 10,000 nucleated cells) in the bone marrow. [2]. In 2006, the criteria required for MSCs’ definition have been defined as follows: (a) plastic-adherent cells when maintained in standard culture conditions; (b) simultaneous expression of stromal markers (CD29, CD44, CD73, CD90, and CD105), but negative for hematopoietic (CD45 and CD14) or endothelial (CD31 and CD34) markers and HLA-DR surface molecules and (c) capacity to differentiate into adipocytes, osteoblasts and chondroblasts in vitro [3].

Although in small amounts, MSCs are present in all organs and tissues, where they play a role in homeostatic maintenance. In fact, many chronic autoimmune or degenerative diseases such as diabetes, lupus, rheumatoid arthritis or psoriasis, and even syndromes associated with early aging, have been shown to have dysfunction or depletion of MSCs. Multiple in vitro studies have demonstrated that MSCs exert a regulatory effect in basic cellular processes such as inflammation, oxidative stress, angiogenesis, etc. Consequently, in vivo studies have demonstrated their efficacy in many animal models (for review, [4]). Furthermore, MSCs are good candidate for cell therapy considering their immune privileged status that allows them to evade immune rejection. Clinical trials have demonstrated the safety and efficacy (phase III clinical trials) of MSCs-based cell therapy for lupus, graft-versus-host disease, diabetes, myocardial infarction or perianal fistulas in Crohn's disease (for review, [5]).

Despite the use of MSCs-based cell therapy, it is now assumed that the mechanism of action of MSCs is basically paracrine. MSCs do not survive long in the body once administered; but during that time, they secrete a cocktail of factors exhibiting a wide spectrum of biological actions. That MSCs-derived secretome is made up of cytokines, hormones and extracellular vesicles, among others. It is considered that MSCs secretome may represent a therapeutic alternative that will avoid the inconveniences associated with cell therapy such as immune compatibility, tumorigenicity or transmission of infections, among others [6].

Regarding cancer, there are controversial data about the role of MSCs, pointing to either tumor promotion or anti-tumorigenic functions in several types of tumors. This points to the importance of MSCs and cancer heterogeneity, as the effects of MSCs on cancer biology could differ depending on the source of MSCs and the type of tumor. This review addresses the current knowledge on MSCs in tumors development and progression, their biological influence and also explores the potential therapeutic strategies for cancer based on MSCs and MSCs-derived products.

## Pro-tumor and anti-tumor effects of MSCs

The tumors were described by Devorak as a “wound that never heals” [7]. In this microenvironment scenario, cancer cells produce a cocktail of growth factors (EGF, HGF, SCF, IGF-1, VEGF, PDGF or βFGF), cytokines (IL-1β, IL-8, TNF-α or TGF-α) and chemokines (CCL2, CCL5, CCL22 or CXCL12) that induce the recruitment of MSCs to the tumor locations, as it has been shown for different types of cancer such as breast, ovarian, gastric, pancreatic, colorectal, skin cancer or melanoma (for review, [8]). Although the exact mechanism for this recruitment remains still unknown, several studies have shown that exogenous MSCs have the ability to migrate into injured tissues, including tumors, up to almost one day after intravenous injection [9]. Literature shows divergent data regarding the anti-tumoral potential of MSCs depending on their tissue origin and the tumor type (Tables 1 and 2).

**Table 1:** Pro-tumor effects of MSCs on the biology of different types of tumors

MSC source	Product administrated	Tumor type	Type of study	Outcome effect	References
Bone marrow	Cells	MDA-MB-231 breast cancer cells	In vitro In vivo	Increase metastasis/activation of the hypoxia-inducible factors	[10]
		MDA-MB-231 and MCF7/Ras breast cancer cells	In vitro In vivo	Promotes breast cancer invasion, epithelial-to-mesenchymal transition and metastasis. Promote de novo production of lysyl oxidase (LOX)	[11]
		HT-29 colorectal cancer cells	In vitro In vivo	Promoted tumor sphere formation and tumor initiation/activation of Janus kinase 2-signal transducer and increased of IL-6 secreted by MSCs signaled through STAT3	[12]
		4T1 mouse mammary tumor cell line	In vitro	Increased tumor growth. Protect breast cancer cells from immune clearance, MSC suppressed the proliferation of PBMC. Inhibition of PBMC migration toward breast cancer cells	[13]
		BxPC3 pancreatic cancer cells	In vitro In vivo	Increase tumor invasion. Increased secretion of MMP-3, amphiregulin and its receptor EGFR	[14]
	Extracellular vesicles	MG63 osteosarcoma cancer cells and SGC7901gastric cancer cells	In vitro	Foster cell growth. Activation of Hedgehog signaling pathway	[15]
Adipose tissue	Cells	MCF-7 breast cancer cells	In vitro In vivo	Stimulate migration and invasion/secretion of IL-6	[16]
		MCF-7 and MDA-MB-231 breast cancer cells	In vitro In vivo	Promote tumorigenesis and angiogenesis/bidirectional signaling; ADSCs differentiated into cancer-associated myofibroblasts	[17]

**Table 2:** Anti-tumor effects of MSCs on the biology of different types of tumors

MSC source	Product administrated	Tumor type	Type of study	Outcome effect	References
Bone marrow	Cells	HepG2 and Huh7 hepatocarcinoma cell	In vitro In vivo	Inhibition of cancer cell proliferation/inhibition of AKT/FOCO3a pathway	[18]
		Primary human glioma cells, HUVEC endothelial cells	In vitro	Reduction in tumor volume and vascular density/reduced expression of platelet-derived growth factor (PDGF)-BB and interleukin (IL)-1β	[19]
		Colorectal cancer cells	In vivo	Administration of MSCs increase the life span of carcinogen-exposed rats by attenuating both colorectal cancer initiation and progression, mediated polarization of resident immune cells which in turn interferes with tumor growth. After fractionated irradiation, MSCs inhibited residual tumor growth, protected healthy tissue and prolonged animal survival	[9]
	Conditioned medium	MDA-MB-231 breast cancer cells	In vivo	Suppressed tumor growth and lung metastasis. Reduced proliferative activity of cancer cells	[20]
		Non-small-cell lung carcinoma cells	In vitro	Inhibition of cell proliferation, viability and migration. Downregulation of mitogen-activated protein kinase (MAPK) signaling pathway	[21]
	Extracellular vesicles	Liver Carcinoma Kaposi’s sarcoma Ovarian tumour cell lines	In vitro	Inhibit proliferation and promote apoptosis	[22]
Adipose tissue	Exosomes	A2780 and SKOV-3 ovarian cancer cells	In vitro	Inhibed proliferation of ovarian cancer cells. Upregulates proapoptotic molecules, induced apoptosis signalling by upregulating different pro-apoptotic signalling molecules (BAX, CASP9, and CASP3), as well as downregulating the anti-apoptotic protein BCL2. Sequencing of exosomal RNAs revealed a rich population of microRNAs (miRNAs), which exhibit anti-cancer activities by targeting different molecules associated with cancer survival, blocking the cell cycle, and activating mitochondria-mediated apoptosis signalling	[23]
Adipose tissue	Cells	U87MG or GSC1 cells Glioblastoma cells	In vitro In vivo	AD-hMSCs showed remarkable tropism towards the tumor, reduction in tumor growth, tumor cell proliferation, and microvascular density	[24]
	Conditioned medium	PC3M-luc2 prostate cancer cells	In vitro In vivo	Inhibit proliferation and promote apoptosis. Reduced the expression of caspase 3/7 with increased antiapoptotic protein, BclxL; effects at least mediated by miR-145 form exosomes released from ASC	[25]
	Extracellular vesicles	Prostate cancer Ovarian cancer Glioblastoma	In vivo In vitro In vivo	Inhibit proliferation and promote apoptosis	[23–25]
Endometrial tissue	Cells	Ovarian cancer cell lines (SKOV3 cells and HO-8910 cells)	In vitro In vivo	Attenuate tumor growth. Induce cell cycle arrest, promote apoptosis, disturb mitochondrial membrane potential and decreasing pro-angiogenic ability	[26]
Uterine cervical tissue	Conditioned medium	MCF-7 and MDA-MB-231 breast cancer cells	In vitro In vivo	Induce cell cycle arrest, promote apoptosis, inhibited proliferation of cancer associated fibroblasts, inhibited macrophage activation, inhibit tumor cell invasion	[27]
Amniotic fluid	Cells	SKOV3 ovarian cancer cells	In vitro	Cytotoxic effect on SKOV3 and suppressed their proliferation; induction of internal and external pathways of apoptosis. Release soluble factors which cause an efficient anticancer effect; induction of its anticancer effects by stimulating the caspase cascade (caspase 3 and 8) and apoptosis. Activation of genes responsible for apoptosis (P53 and P21)	[28]
Placental chorionic villi	Cells	MDA-MB-231 breast cancer cells	In vitro	Reduce the proliferative and migratory capacity of tumor cells, inhibited the endothelial cell-associated vasculogenic capacity	[29]
Umbilical cord	Cells	Line MDA-MB-231 human breast cancer cells	In vivo	Antitumor effect. Inhibited tumor angiogenesis and induced cell apoptosis	[30]
		Liver cancer cell lines HepG2 and SK-Hep-1	In vitro In vivo	Reducing hepatoma cell growth and metastasis. Downregulation of Wnt/β-catenin signaling pathway	[31]
		High-grade human glioma cell lines (SNB19 and U251) and xenograft cell lines (4910 and 5310)	In vitro In vivo	Inhibited tumor growth. Upregulation of PTEN gene phosphatase and tensin homolog deleted on chromosome 10 (PTEN) in tumors induced cellular death through decreasing XIAP expression	[32]
	Conditioned medium	MCF-7 tumor cells	In vitro	Cytotoxic effects on MCF-7 cells by induction of apoptosis	[33]
		Ovarian cancer cell lines (OVCAR3 and SKOV3)	In vitro	Reduction in size of tumor spheres. Increase in the sub-G1 and G2M phases of cell cycle, inhibition of cell migration, activated caspase; decreased expression of cell cycle regulatory genes (cyclin A2, Cyclin E1), prostaglandin receptor signaling genes (EP2, EP4) and the pro-inflammatory genes (IL-6, TNF-α); cycle arrest, apoptosis	[34]
		MG-63 and SKES-1 osteosarcoma cell lines	In vitro In vivo	Decreased tumor sizes/Inhibition of mammary carcinoma and osteosarcoma cells via apoptosis and autophagy	[35]
		HeLa cells	In vitro	Reduced cell viability and increased apoptosis. Increased caspase-3/7 activity, decreased mitochondrial membrane potential, and induced cell cycle arrest	[36]
		MDA-MB-231 breast cancer cells	In vitro In vivo	Suppresses breast cancer cells growth and sensitizes cancer cells to radiotherapy. Inhibition of the Stat3 signaling pathway	[37]

### Pro-tumor functions

Among the proposed mechanisms for MSCs contributing to tumor progression are: (i) Promotion of increased function and count of tumor stroma cells, (ii) Promotion of angiogenesis (iii) Suppression of the immune response to tumor, (iv) Enhancement of tumor cell survival, cancer cell aggressiveness and tumor metastasis and (v) Enhance of drug resistance.

#### Promotion of increased function and count of tumor stroma cells

MSCs show the ability to differentiate into different cell types of the tumor stroma, which in turn, have the ability to contribute to tumor progression, such as cancer associated fibroblasts (CAF), cancer associated adipocytes (CAA), pericytes or endothelial-like cells.

CAF, which differ from normal fibroblasts by presenting a different gene expression profile and promoting cancer cell aggressiveness [38], are one of the most abundant cell types in the cancer stroma of human tumors. MSCs have been shown to have a great ability to differentiate into CAF in the TME compared to non-neoplastic tissues [39]. This may be due to the factors released by cancer cells, that would induce the activation of the TGF-β/Smad signaling pathway [40].

Among the different mechanisms by which CAF promote tumor progression are the following: (i) contractile forces exerted by CAF that can alter the basement membrane, facilitating cancer cell invasion; (ii) production of metalloproteases inducing the degradation of the extracellular matrix (ECM); (iii) angiogenic promotion; (iv) epithelial–mesenchymal transition (EMT) activation; (v) metabolic reprogramming toward a reverse Warburg phenotype; (vi) secretion of key biological factors (such as cytokines: IL-1β, IL-6, IL-8; growth factors: SDF-1, FGF, HGF; and NFκB) to induce immune cell recruitment that may contribute to tumor progression, (vii) induction of resistance to cancer therapy (for review, [1, 41]).

Similarly, CAA also differ from the normal adipocytes in their high metabolic activity and in their ability to generate a variety of growth factors, hormones, cytokines and adipokines, which induce tumor growth, metastasis and therapy resistance (for review, [41]). In addition, adiponectin, which plays an anti-tumorigenic role by inducing apoptosis, is decreased in CAA [42].

#### Promotion of angiogenesis

Due to the high demand of oxygen and nutrients by cancer cells, the development of new blood vessels from existing vasculature is necessary to sustain either the early steps of tumor development as its progression [43]. In vitro studies reported that different MSCs populations induce the proliferation and migration of endothelial cells, promoting tube formation and preventing endothelial cell apoptosis [44].

Experimental results indicate that tumor growth promotion in vivo by MSCs may be attributable, in part, to enhanced angiogenesis [45]. MSCs can increase angiogenesis through the induction of ERK1/2 and p38 MAPK pathways, which enhance the expression of VEFG and CXCR4 in tumor cells [46]. MSCs also contribute to tumor angiogenesis through their potential to differentiate into endothelial-like cells and/or pericytes [47, 48]. In addition, it has been shown that MSCs secrete pro-angiogenic soluble factors such as VEGF, PDGF, angiopoetin, LIF, M-CSF, MIP-2, IL-6, IL-8, TGF-β, IFN-γ, β-FGF and TNFα [16, 49–52]. Otherwise, angiogenic inhibitors have been also identified in the MSCs secretome [53]. Nevertheless, the secretion of these pro- and anti-angiogenic factors by MSCs, can be modified by regulating several factors such as hypoxic conditions, which is a common condition in tumors [54].

#### Suppression of the immune response to tumor

MSCs are key regulators of innate and adaptive immune responses and possess strong immunosuppressive properties, which would support the potential evasion of tumor cells from anti-cancer immunity [55]. MSCs within the TME could induce immunosuppression mainly by the secretion of soluble factors and mediators such as cytokines (TGF-β, HGF, IFN-γ, TNF-α, IL-1α, IL-1β, IL-4, IL-6 and IL-10), prostaglandin E2 (PGE2), HLA-G, nitric acid, indoleamine 2,3-dioxygenase (IDO) and prostaglandin E2 indoleamine, as well as by their interactions with various immune cell types. [55–59]. Table 3 shows pro-tumor effects of MSCs on several immune cells, such as neutrophils, dendritic cells, natural killer cells, T cells, B cells, macrophages and myeloid-derived suppressor cells.

**Table 3:** Immunosuppressive effects of MSCs on several immune cell types which contribute to tumor progression

Immune cells	MSCs effects	References
Neutrophils	Induction of CD11b/Ly6G-positive neutrophils to massive T-cell inhibition in vitro, and enhancement of breast carcinoma tumor growth in vivo IL-6 from cancer-derived MSCs promotes neutrophil activation via STAT3-ERK1/2 signaling and induces their polarization towards a tumor-supportive phenotype in gastric cancer	[60, 61]
Dendritic cells	Suppression of dendritic cell differentiation by downregulating IFN-γ and TNF-α expression Regulation of maturation of dendritic cells via PGE2 signalling Promotion of immunosuppressive effects on dendritic cells and tumor growth in murine melanoma tumor models	[62–64]
Natural killer	Block its activity, suppressed its proliferation and cytokine secretion and reduce its ability to produce IFN-γ	[65, 66]
T-cells	Repress T-cells proliferation and increase apoptosis by secreting soluble TGF-β Secrete IDO, which inhibits T-cells through tryptophan depletion	[13, 67–70]
B-cells	Repress B-cell proliferation by secreting soluble factors Reduce antibody production and inhibit their differentiation to plasma cells Attenuate B-cell proliferation and antibody production by INF-y stimulated-MSCs overexpression galectin-9	[70–73]
Macrophages	Induce macrophages to produce the anti-inflammatory factor IL-10 Decrease the phagocytic abilities of macrophages, thereby promoting a pro-tumorigenic macrophage phenotype by secreting soluble factors	[74–76]
Myeloid-derived suppressor cells	Protect against autoimmunity by recruitment of myeloid-derived suppressor cells via CCL2 signalling	[77]

#### Enhancement of tumor cell survival, cancer cell aggressiveness and tumor metastasis

MSCs have been found to release many soluble factors that promote tumor survival and its proliferation, including VEGF, FGF-2, PDGF, HGF, BDNF, SDF-1α, IGF-1, IGF-2, TGF-β, and IGFBP-2 [78–80].

Epithelial–mesenchymal transition (EMT) is a process in which epithelial cells undergo multiple changes to gain mesenchymal properties. EMT is a relevant phenotypic change allowing cancer cells to detach from the primary tumor, being the initial step in metastatic spread. This process implicates the activation of several transcription factors (Snail, Slug, Twist, and FOXC2) [81] and a decreased E-cadherin expression [82]. Paracrine signals of MSCs, via secretion of growth factor or cytokines (EGF, HGF, PDGF or TGF-β) may induce these EMT-specific transcription factors [83, 84]. In addition, it has been shown that MSCs within the tumor stroma of breast cancer enhance EMT by producing CCL5 (also called RANTES). CCL5 increases the secretion of MMP-9, which degrade basal membrane and extracellular matrix, promoting cancer cell motility and metastasis [85].

Nowadays, it is assumed that cancer stem-like cells (CSC) represent a cell subpopulation of tumors responsible for cancer initiation, and also in charge of the final steps of colonizing premetastatic niches and chemotherapy resistance [86]. Experimental evidence indicates that MSCs increase stemness of cancer cells by secreting factors capable of activating pathways such as JAK2 / STAT3 in lung cancer cells [87];secreting CXCL7 [88] and increasing P2X-mediated signaling [89] in breast cancer cells; potentiating WNT and TGF-β signaling pathways in gastric cancer [90]; activating the Hedgehog/BMP4 signaling loop in ovarian cancer [91]; and via IL-8/mitogen-activated protein kinase (MAPK) in colorectal cancer [92]. Interestingly enough, it has been also reported that gastric mucosal cells, after being infected with *Helicobacter pylori*, recruit MSCs to the site of the infection and then they differentiate into gastric cells expressing epithelial markers (such as KRT1-19 and TFF2), which together with chronic inflammation, could promote a CSC phenotype of gastric cancer [93].

MSCs are involved in other mechanisms critically contributing to the metastatic process, either at the primary tumor or at pre-metastatic sites. MSCs secrete TGF-β, which increases cancer cells’ invasive and migratory potential [94]. In addition, they secrete chemoattractants such as CCL5, CXCL1, CXCL5, CXCL7 and CXCL8 and CXCL12, which induce migration of tumor cells to metastatic lesions [85, 95]. Among these factors regulating the trafficking of tumor cells, CXCL12 seems to be especially relevant, as metastatic cells express its major receptor, CXCR4 [96, 97]. MSCs have also been proved to support metastatic niches due to their strong adhesive activities, mediated by adhesion molecules and integrins [98].

#### Promotion of drug resistance

Tumor resistance to chemotherapy remains one of the major obstacles of modern clinical oncology. MSCs may contribute to drug resistance as there are evidence illustrating this effect, although the exact mechanisms are not entirely known. As discussed above, MSCs might promote drug resistance through activation of EMT, CAF and especially, CSC. CSC represent a unique, rare population of cells within tumors that resist to many cytotoxic agents using several mechanisms, such as their low proliferative rate, their high DNA repair capabilities and their expression of membrane transporters to control a cytotoxic drug influx [99].

Curiously, MSCs may be recruited in large number to tumors in response to chemotherapy helping cancer cells develop the resistance to the therapy [100]. In addition, there are evidence indicating that pre-exposure of MSCs to chemotherapeutic agents alters phosphorylation levels of several tyrosine kinases (WNK-1, c-Jun, STAT3 and p53), favoring MSCs survival and stimulating their production of cytokines, which would promote chemoresistance of tumor cells [101].

Paracrine secretion of IL-6 by MSCs may induce chemoresistance to cisplatin in thymus residing endothelial cells in mice [102], or to paclitaxel in head and neck carcinoma [103]. It also been pointed that activation of CXCL12-CXCR4 axis in MSCs reduces imatinib-induced cell death in chronic myeloid leukemia [104], or protect cancer cells from hyperthermia-induced cell death induced by intraperitoneal chemotherapy in ovarian cancer cells [105]. In pancreatic adenocarcinoma, MSCs increased chemoresistance to gemcitabine by activating the CXCL10-CXCR3 axis [100]. Other protective-drug activities of MSCs were described, for example, increasing RNA and protein synthesis against the cytotoxic effects of forodesine in chronic lymphoid leukemia [106], or by secreting polyunsaturated fatty acids (12-oxo-5,8,10-heptadecatrienoic acid [KHT], and hexadeca-4,7,10,13-tetraenoicacid [16:4(n-3)]) which may have an indirect protective effect against cisplatin [107].

### Anti-tumor functions

In addition to the pro-tumorigenic effects described above, other studies have shown that MSCs act in an anti-tumorigenic manner suppressing disease progression. Studies, both in vivo and in vitro, have explained that MSCs can inhibit tumor growth and metastasis through several mechanisms such as:

(i) Regulation of cellular signaling pathways and induction of apoptosis, (ii) Inhibition of angiogenesis (iii) Modulation of immune response.

#### Regulation of cellular signaling pathways and induction of apoptosis

MSCs can attenuate cancer cell proliferation by paracrine inhibition of cell signaling pathways in several types of tumors, such as breast, ovary, stomach, colon, liver and skin. These mechanisms include the inhibition or suppression of phosphoinositide 3-kinase PI3K/AKT, and WNT/β-catenin signaling pathways [108–110]. MSCs from several sources have been also reported to induce tumor cell apoptosis. MSCs from gingival tissue induce cell death of oral cancer cells [111]. Also, it has been reported the induction of apoptosis of breast cancer cells by adipose derived (AD)-MSCs through IFN-γ induction [112], and MSCs from the human uterine cervix (hUCESC) are capable of inducing death of cancer and stromal cells through caspase 3 and annexin V activation [27].

#### Inhibition of angiogenesis

Although, as described above, there is evidence indicating proangiogenic effects of MSCs, there are also data indicating that, under certain circumstances, some types of MSCs can exert an anti-angiogenic effect. Thus, umbilical cord (UC)-MSCs have been described to inhibit angiogenesis as in vitro as in vivo in gliomas, which is accompanied by a downregulation of pro-angiogenic factors (PDFG-BB, IGF-1, FGF-2, and IL-1β) [19]. It has also been described that human placental chorionic villi-derived MSCs inhibited the endothelial cell-associated vasculogenic capacity of the breast cancer cell line MDA-MB-231 on HUVEC cells [29]. These data suggest that the involvement of MSCs in angiogenesis is highly regulated and further studies will be required to fully understand it.

#### Modulation of immune response

It is widely accepted that MSCs tend to be more pro-tumorigenic than anti-tumorigenic because of their immunosuppressive and regenerative activities [113–115]. Nevertheless, there is other evidence indicating the possibility of an MSCs-induced anti-tumor modulation of the immune system in the TME. It has been reported that the immortalized mesenchymal progenitor cell line MPC1cE increased monocyte and granulocyte infiltration in tumors, inhibiting cancer growth in a rat colon cancer model [116]. In addition, there are data indicating that bone marrow (BM)-MSCs can induce changes in the immune cell phenotypes towards an anti-tumor behavior, such as altering the ratio of Treg and myeloid-derived suppressor cells to CD8+ T cells [117], increasing neutrophil function through Toll-like receptor 3 (TLR3) activation [118, 119], as well as hUCESC inhibiting and reverting macrophage differentiation [27]. Interestingly, in a recent study in a colorectal cancer model in immunocompetent rats, BM-MSCs were able to modulate the inflammatory response during the early phase of chemically-induced carcinogenesis. Locally, mRNA levels of several proinflammatory genes, including IL-1β, IL-6, TNF-α, and MIP-2, were significantly downregulated, whereas mRNA levels of the anti-inflammatory genes IL-10 and TGF-β were upregulated. Even more, a polarization of resident macrophages into the M1 subtype was observed [9].

It is also important to note that CAF could mediate inflammation and by recruiting macrophages stimulate angiogenesis, which may promote tumor growth [120]. In this regard, it has been showed that hUCESC reduce cell proliferation, increase apoptosis and decrease invasion capacity of CAF [27].

#### MSCs tumor or anti-tumor effects depending on MSCs origin and tumor type

The effects of MSCs on tumors are still controversial [115, 121, 122], although it is widely accepted that MSCs tend to be more pro-tumorigenic than anti- tumogenic, by being immunosuppressive. However, based on the accumulated information during the last decade, it is time to accept a new scenario in which the pro-tumor or anti-tumor effect of MSCs depends on the source of MSCs and the type of tumor. In fact, the most heterogeneous effects of MSCs, according to their origins, are those occurring on tumors [123, 124]. As can be observed in Tables 1 and 2, we can conclude that BM-MSCs and their secretome derived products have pro-tumor effects on pancreatic and gastric cancer cells, whereas they have anti-tumor effects against glioma cells and non-small-cell lung carcinomas, and there are conflicting results about the effect of these cells on breast and colorectal carcinomas. In regard to AD-MSCs, anti-tumor effects were found in ovarian, prostate and glioblastoma cancer cells, but discordant results are indicated for breast cancer cells. Also, it is important to note the different behavior and effects in tumors depending on the use of cells or their secretome-derived products. Thus, it has been shown that secretome-derived products of BM-MSCs, such as conditioned medium or exosomes, are able to reduce proliferation and migration and increase the apoptosis of certain types of cancer cells, such as non-small-cell lung carcinoma, liver carcinoma, Kaposi’s sarcoma, and ovarian tumor cell lines. The same effect was reported for extracellular vesicles (EVs) from AD-MSCs, that inhibit prostate cancer, ovarian cancer cells, or glioblastoma (Table 2).

On the other hand, relevant and consistent results are found regarding MSCs from uterine origin. In all studies, including MSCs from endometrial, uterine cervical, as well as other reproductive tissues (amniotic fluid, placental chorionic villi and umbilical cord), potent anti-tumors effects have been found against breast, ovarian, liver, glioma or osteosarcoma cancer cells. These data may indicate that MSCs derived from reproductive tissues could have a genuine anti-tumor effect. For example, it has been indicated that UC-MSCs have a high tendency to move toward tumor cells and inhibit the growth of solid tumor cells such as breast [33, 34] or HeLa cells [35, 36]. The unique feature of these cells leads to the hypothesis suggesting that UC-MSCs may act as a natural defense against the migration of cancer cells from the mother to the fetus, justifying why tumors in fetus are very rare [125]. In any case, these functional particularities of MSCs according to their anatomical location, allow us to consider the existence of MSCs with special capacities pursuant to their biological environment. In this context, it is reasonable to consider the existence of MSCs accustomed to regulate homeostasis in tissues highly exposed to external aggressions. One location that appears to be candidate for hosting this kind of special MSCs is the uterine cervix.

The human uterine cervix is permanently in contact with a mildly aggressive environment (bacteria and acid pH) that may become overly pathogenic (infection by fungi or some strains of the papillomavirus family). At this location, a process known as “squamous metaplasia”, by which the glandular cells of the endocervical epithelial lining convert into squamous epithelial cells of the ectocervix, take place. This process is a biologically very risky process, because it implies a regression of the original transforming cell into a highly undifferentiated state, from which it can redifferentiate into the new kind of cell required. For one thing, a highly undifferentiated cell is prone to uncontrolled hyperproliferation, which is the first step in the oncogenic transformation process. For another thing, it is more vulnerable to the infection by viruses, in this case most notably by the oncogenic variants of the papillomavirus family, due to a higher DNA replication activity. Similarly, on breast cancer, hUCESCs do indeed allow a certain degree of hyperproliferation, so that they do not act on the non-invasive, low-proliferating MCF-7 cell line. Notwithstanding, once a critical threshold is reached, they exert a very potent and effective anti-tumor activity against highly proliferating and metastasis-producing cancer cells, such as the MDA-MB-231 breast cancer cells tested. All these effects were reproduced in an almost identical fashion on cell lines produced from samples obtained in the clinic from real patients [27]. Therefore, our hypothesis is that hUCESC, a very special kind of MSCs embedded in the uterine cervical stroma, have a unique ability to hold all these extreme biological risks under control, by the regulation of proliferation and oncogenic transformation [27], inducing apoptosis if ultimately necessary, [27] and also exerting anti-inflammatory [126], antibacterial [127] and antifungal effects [128] through, among other mechanisms still under study, the secretion into its culture medium of a highly complex cytokine cocktail mediating all of the just mentioned effects,as we shall see hereunder.

## Role of the MSCs in the “alliance” and “war” of the intercellular signals from tumor microenvironment

**Fig. 1:**
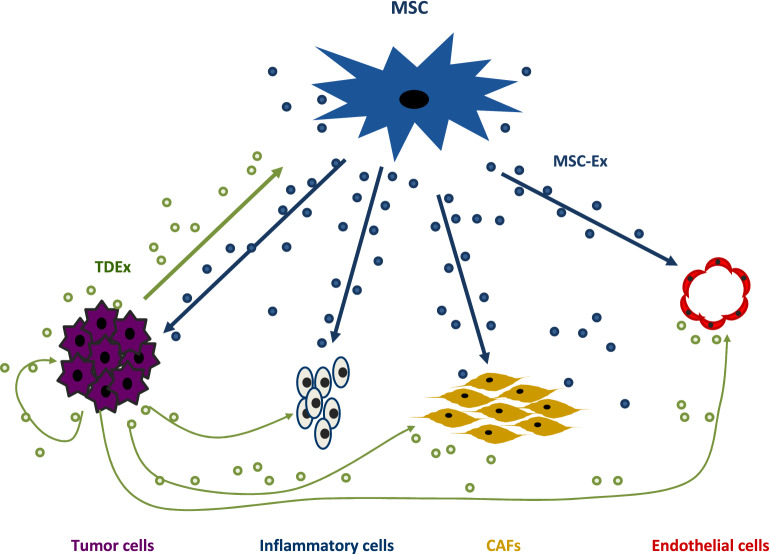
Scheme of relationships established by the MSCs with different cell-types during tumor progression

We can consider tumor growth as the result of the "alliance" or "war" of intercellular signals between the different types of cells in the tumor scenario. Schematically, cancer cells secrete cytokines and chemokines, such as TGF-β and CCL2, involved in the recruitment and activation of CAF [129] and mononuclear inflammatory cells, as well as the tumorigenic transformation of macrophages [130]. Furthermore, after recruitment of stromal cells, a complex and dynamic interaction takes place [1, 131]. In this context, MSCs seems to play a pivotal role, by interacting with cancer cells and stromal cells (Fig. 1). The multi-directional signals between these cells are mediated by soluble factors, integrins, and/or EVs (for review [1]) (apoptotic bodies, microvesicles and exosomes), resulting in a particular nano-communication among the different cell types of the tumor [132]. Exosomes, the smallest subset of EVs (30–150 nm in diameters), are enclosed by a protein-phospholipid bilayer membrane, and its lumen recapitulates, partially, the content of the parent cell (DNA, messenger RNA (mRNA), microRNA (miRNA), nucleic acids, growth factors, cytokines and chemokines) [133, 134].

Tumor cells and MSCs are important producers of exosomes. Tumor-derived exosomes (TDEx) are ubiquitously present in the tumor environment (TME) and in body fluids of patients with cancer [135]. TDEx, whose content depends on the type of tumor cell [136], transmit messages from the parent tumor cell to normal or malignant cells in the TME, including messages to MSCs [137]. These messages to stromal cells and MSCs include the overexpression of genes involved in cell migration, matrix remodeling, angiogenesis and tumor growth [138–140].

Under normal physiological conditions, MSCs also produce high dose of exosomes (MSCs-Ex) [141]. MSCs-Ex carry a complex cargo of molecules including more than 850 unique gene products and more than 150 different miRNA [142, 143]. Thus, MSC-Ex have the potential to elicit different cellular responses in a broad variety of cells [144], which seem to be responsible for the modulation of many physiological functions [145, 146]. MSCs are recognized as recipients for signals from the tumor, but through MSCs-Ex, MSCs have the capacity to interact with multiple cell types in the TME inducing phenotypic and functional changes which may exert profound effects on tumor growth [147]. In a similar way to MSCs, it has been reported that some types of MSCs-Ex can induce and support tumor growth, invasion and metastasis[144, 147, 148], while other MSCs-Ex have anti-tumor actions, depending on their provenance and the type of tumor. For example, it has been found that exosomes derived from MSCs can have an antiproliferative effect and even induce a state of latency in some tumors such as mammary or ovarian tumors [149, 150]. Exosomes obtained from BM-MSCs were also found to inhibit proliferation and promote apoptosis in liver carcinoma, Kaposi's sarcoma, and ovarian tumor cell lines [151] and exosomes derived from AD-MSCs inhibit prostate cancer [25], ovarian cancer cells [23], or glioblastoma cells [24]. In addition, it has been reported that MSCs-Ex from human UC-MSCs attenuate the growth of bladder carcinoma cells, possibly by down-regulating phosphorylation of Akt protein kinase and up-regulating cleaved caspase-3 [152].

Similarly, intratumoral injection of miR-146b-expressing MSCs-Ex resulted in a considerable reduction in glioma xenograft development in a brain tumor rat model by decreasing the growth, migration and invasion of cells [153].

Presently, it is known that one of the antitumor actions of exosomes is largely due to miRNA contained inside. miRNAs are small non-coding RNAs that suppress the translation and stability of mRNA, controlling in this manner several cellular processes, such as cell cycle regulation, cell differentiation and apoptosis. Dysregulation of miRNA has been revealed to play an essential role in the development of tumor progression [154]. In the context of the heterogeneity shown by the MSCs, different populations of exosomes containing miRNA with anti-tumor effects have been described, for example: miRNA-9-3p from exosomes secreted by BM-MSCs suppresses the development of urinary bladder cancer [155], miRNA-143 derived from that same type of MSCsinhibits cell migration and invasion of prostate cancer [156], miRNA-124 derived from UC-MSCs increases chemosensitivity to temozolomide and decrease migration from glioblastoma cells [157], and miRNA-122 derived from MSCs of adipose tissue induces hepatocarcinoma cancer cells to be more sensitive to the chemotherapeutic agent sorafenib [158]. Furthermore, miRNA-379, derived from BM-MSCs, has also recently been shown to induce a decrease in tumor activity and size in breast cancer [159].

The outcome of tumor progression is highly dependent on the outcome of those signal balances, and cancer cells have been demonstrated to internalize higher percentage of exosomes than normal cells [160, 161]. Then, these EV produced by MSCs can be responsible for many of their anti-tumor effect. Therefore, the use of MSCs with anti-tumor capacity may signify a great opportunity for a therapeutic strategy.

## MSCs as therapeutic strategies in tumors

### MSCs as cell therapy

As described above, certain MSCs, according to their origin, and especially those from reproductive source, seem to have an anti-tumor effect for specific carcinomas. This, together with the tropism that MSCs show for tumors makes them potential candidates to be applied in future clinical trials. However, it is important to mention that regarding their affinity for tumors, MSCs are being developed as selective vehicles for drug delivery, especially in aggressive neoplasms. For example, MSCs have been used to deliver oncolytic viral loads into tumors, therefore selectively inducing cancer cell killing, preclinically, and most recently, also clinically [162, 163]. In this same context, MSCs have been also genetically manipulated to express immunodulatory cytokines or specific enzymes, which can promote cancer cell killing effects. It has been presented that MSCs overexpressing IL-12 enhance anti-tumor T cell responses and decrease tumor growth [164]. MSCs genetically modified to produce IFN-β induce significant anti-proliferative effects in melanoma cells [165] and in a metastatic prostate cancer preclinical model [166]. In addition, MSCsoverexpressing TNF-related apoptosis-inducing ligand (TRAIL), can effectively eliminate cancer cells in several cancer models including glioma [167], pancreatic [168] and lung cancer [169, 170].

On the other hand, MSCs have been also genetically manipulated to express specific enzymes, such as cytosine deaminase or herpes simplex virus-thymidine kinase (HSV-TK), which convert inactive systemically administrated prodrugs, like fluorouracil and ganciclovir, into active cytotoxic agents [171, 172], offering the advantage to increase tumor-directed chemotherapy activity and to minimize systemic toxicity.

However, currently, there are problems derived from using stem cells themselves, such as immunological incompatibility, tumorigenicity, embolus formation, transmission of infections, and the potential entry of MSCs into senescence [173]. In addition, there are other potential drawbacks related to the expected clinical benefit of MSCs cancer therapy, as their physiological differentiation into mesenchymal lineages that may decrease therapeutic potential [174].

### MSCs secretome

Secretomes, aside from avoiding the inconveniences of administering living proliferating cells, show other additional advantages. Unlike cell therapies, secretomes can be better evaluated for their safety, dosage and potency, analogously to conventional therapeutic agents. Secretomes can be stored without the application of potentially toxic cryopreservative agents. The use of products derived from the secretome, such as the conditioned medium or extracellular vesicles (EVs), is cheaper and more practical for clinical use, since the use of the secretome could avoid the time and costs associated with the expansion and maintenance of clonal cell lines. This is due to the fact that the secretome for therapies could be prepared in advance in large quantities and kept available for treatments when necessary.

#### MSCs conditioned medium

It has been disclosed that MSCs secrete high amounts of cytokines, which induce inhibition of tumor growth, such as IFN-α, IFN-β, IFN-γ, DKK-1/3, IL12, TRAIL (Tumor Necrosis-Factor-Related Apoptosis-Inducing Ligand), tumor necrosis factor superfamily member 14 (TNFSF14) also known as LIGHT, Fms-related tyrosine kinase 3 (FLT-3) ligand, C-X-C motif chemokine 10 (CXCL10) and liver-enriched transcriptional activator protein (LAP) [27, 30, 165, 175–178]. It has been also reported that the anti-tumor effect of MSCs may be partly related to the activity of the tissular inhibitors of matrix metalloproteinase (MMPs) TIMP-1 and TIMP-2, present in their secretome [178, 179], being the inhibition of MMPs associated with the inhibition of migration and invasion of cancer cells.

Interestingly, paracrine factors, collectively named as secretome, are estimated to be responsible for up to 80% of the therapeutic effect of MSCs. The heterogeneity of secretomes with respect to the tissue origin of MSCs is known. In this regard, we previously reported that hUCESCs-derived secretome produces higher antitumor cytokines than thoses from AD-MSCs. These antitumor citokines includes LIGHT, FLT-3, CXCL10 and LAP. On the contrary, several markers of tumor progression, such as epithelial growth factor receptor (EGFR), fibroblast growth factor (FGF), intercellular adhesion molecule 3 (ICAM3), interleukin-6 (IL-6), c–c motif ligand 7 (CCL-7), macrophage migration inhibitory factor (MIF), soluble glycoprotein 130 (sgp130) and vascular endothelial growth factor D (VEGF-D) are not detected or are lower in hUCESCs comparedto the secretome of AD-MSCs (23). These differences in the composition of the secretomes may partially explain the different anti-tumor or pro-tumor effects of the MSCs according to their tissue origen. In this context, we may also consider that the anti-tumor acivity of AD-MSCs can be to a certain extent clarified by their high amounts of pro-angiogenic molecules [180] and MMPs [181] which secrete, whereas hUCESCs segregate large amounts of TIMP-1 and TIMP-2 [182].

However, the use of conditioned medium can have inconvenients, such as its scalable production, high salt content or being a poorly defined product, which can be a barrier for authorization by regulating agencies. An alternative for these problems could be the use of the EVs or exosomes present in these media.

#### MSCs extracellular vesicles

In general, it is assumed that MSCs-derived EVs perform similar functions to parent cells [183]. However, there are few studies on the basis of which we can evaluate the pro-tumor or anti-tumor effects of MSCs-derived EVs in accordance to their parent MSCs and pursuant to their different origins. In these few studies, for example, tEVs derived from BM-MSCs were shown to promote the growth of osteosarcoma and gastric cancerous cells (Table 1) [15],and inhibit the proliferation and promote apoptosis in liver carcinoma, Kaposi´s sarcoma and ovarian tumor cell lines (Table 2) [22]. Meanwhile, EVs derived from AD-MSCs were exhibited to inhibit the growth of prostate cancer cells, ovarian cancerous cells and globlastoma Table 2) [23–25].

These findings seem to indicate a complex interaction of MSCs-derived EVs with tumor biology, which depend not only on the tissue origin of MSCs, but also on the type of tumor. Therefore, we must assume that further studies are required to better identify which MSCs produce the most appropriate EVs for each potential antitumor therapy.

Even so, MSCs are the only human cell type known that can be used for the mass production of EVs for drug delivery [184], maintaining the tumor homing ability [185]. EVs, including exosomes, are smaller, less complex and less immunogenic than their parent cells, since they have a lower content of membrane-bound proteins [186]. Furthermore, production and storage of EVs are easier than for their parental cells. In addition, other advantages of EVs include longer circulating half-time [187] or better crossing through the blood–brain barriers [188]. Also, EVs can be easily manipulated and can be modified with certain ligands or proteins on their surface in order to improve their targeting capability [189].

In order to enhance vesicle release from MSCs cells, several strategies have been proposed, such as prolonged culture and maintaining cells at low pH [190, 191]*.* The establishment of immortalized cells from MSCs is another strategy proposed to scale up EVs production [192], and, for instance, the overexpression of the oncogene *c-myc* has been reported to increase EVs production in MSCs [193].

On the other hand, MSCs-EVs have an additional interest for oncological therapy due to their tropism towards tumors, potentially behaving like “Trojan horses”. Thus, it was shown that cancer cells internalize higher percentage of exosomes compared to normal cells [160, 161, 194]. The internalization of exosomes by cancer cells was also found to be 10 times greater than the internalization of liposomes of comparable size [160]. Furthermore, although the mechanism explaining the affinity of exosomes towards tumors is not yet well understood, it is known that the acid pH intratumor condition increases the internalization of exosomes [195], and also tumors are known to harbor a particularly acidic environment. This property of exosomes has raised their interest as carriers of anti-tumor factors.

Many types of “nanocarriers” have been developed in order to achieve the accumulation of anti-tumor agents to target cells (nano-gold particles, carbon nanotubes, molecules and liposomes, polymeric nanoparticles and polymer conjugates). Among those particles, the above-mentioned liposomes are the more effective ones. However, they have the drawback of causing immune rejection. For this reason, natural elements have been tested with tropism towards target cells, such as viruses, bacteria, erythrocytes, macrophages, lymphocytes, stem cells and exosomes. Among all of them, exosomes are the most promising natural carriers, due to their wide distribution by biological fluids and their intrinsic ability to search targets. [196, 197].

Exosomes can also be loaded with particles using different techniques, such as chemical transfection, incubation, electroporation, or by transfection of exosome-producing cells [198]. Regarding anti-cancer therapy, exosomes have been loaded with cytotoxic chemotherapy agents, small interefering RNA (siRNA) or miRNA.

It has been informed that MSCs-EVs loaded with paclitaxel, doxorubicin or gemcitabine reduce cell viability and inhibit oral squamous cancer cell growth [199]. In addition, exosomes loaded with methotrexate and functioned with a synthetic multifunctional peptide have also been shown to facilitate the membrane receptor mediated internalization procedure, both in vitro and in vivo in a glioma model [200]. In this line, it has also been shown that after treating several populations of human MSCs with sub-lethal concentrations of taxol for 24 h, exosomes obtained showed 80% cytotoxicity against human lung, breast, or ovarian cancer cell lines. Likewise, these same microparticles intravenously administered caused a reduction of more than 60% of primary subcutaneous tumors and caused a significant reduction in metastasis in a xenograft model of these tumors in mice [201].

Exosomes transfected with different miRNA have demonstrated anti-tumor effects in different in vitro and in vivo models. For example, exosomes transfected with encapsulated miR-379 have been administered for breast cancer therapy in vivo with positive therapeutic effects [159]. Exosomes from mouse MSCs treated with miRNA-133b suppress glioma progression [202], exosomes from umbilical cord MSCs transfected with miRNA-148-3p slow down breast cancer progression [203], exosomes from BM-MSCs transfected with miRNA-205 prevent the progression of prostate cancer [204], or exosomes from BM-MSCs transfected with miRNA-1231 inhibit the activity of the pancreatic cancer [205].

In line with this, it has also been reported that MSCs-EVs loaded with siRNA can silence genes driving tumorigenesis. For example, MSCs-EX loaded with siRNA for polo-like kinase I decreased bladder cancer cell proliferation [161].

## Conclusions and future perspectives

MSCs appear to play a central role in the context of intercellular signals between cancer cells and tumor stromal cells. We can consider the existence of cancer-educated MSCs that contribute to promote tumor progression, but also the possibility of MSCs not residing in the TME that may be an alternative to anti-tumor therapy. To establish this therapeutic hypothesis, we must start from the evidence indicating that the pro- or anti-tumor effect of MSCs will depend on the origin of the MSCs and the type of tumor. In this sense, the existing data seem to converge on the fact that MSCs originated in the uterus and pregnancy-related tissues have a broader antitumor effect, so they could be good candidates for oncological therapies. Likewise, products derived from the MSCs secretome, such as exosomes, can represent a good therapeutic strategy, avoiding the drawbacks related to direct cellular therapies. An additional advantage of these new biological products is the tropism towards tumors and their suitability to be manipulated in order to increase their effectiveness and potency. However, it is necessary to advance in the investigations that allow us to: (i) establish the most appropriate type of MSCs for each type of tumor; (ii) optimize the isolation and culture methods of these MSCs; (iii) define practical and reproducible methods for obtaining biological products of therapeutic interest derived from the secretome of these MSCs; and, (iv) identify the appropriate functional tests to measure these products prior to their application in patients. To achieve these goals, we must conveniently integrate technologies, such as cell culture techniques that include physical, chemical, biological and genetic manipulation, and the use of bioreactors, as well as the use of sophisticated extracellular vesicle isolation techniques, nanotechnology and artificial intelligence.

## Data Availability

Not applicable.
